# Prevalence, types and demographic features of child labour among school children in Nigeria

**DOI:** 10.1186/1472-698X-5-2

**Published:** 2005-03-02

**Authors:** Bolanle M Fetuga, Fidelis O Njokama, Adebiyi O Olowu

**Affiliations:** 1 Department of Paediatrics, Obafemi Awolowo College of Health Sciences, Olabisi Onabanjo University, Sagamu, Ogun State, P.MB. 2022, Nigeria; 2 Havana Specialist Hospital, Lagos, Nigeria; 3 Obafemi Awolowo College of Health Sciences, Olabisi Onabanjo University, Sagamu, Nigeria

## Abstract

**Background:**

To determine the prevalence, types and demographic features of child labour among school children in Nigeria.

**Methods:**

A cross-sectional interview study of 1675 randomly selected public primary and secondary school pupils aged 5 to less than 18 years was conducted in the Sagamu Local Government Area of Ogun State, Nigeria from October 1998 to September 1999.

**Results:**

The overall prevalence of child labour was 64.5%: 68.6% among primary and 50.3% among secondary school pupils. Major economic activities included street trading (43.6%), selling in kiosks and shops (25.4%) and farming (23.6%). No child was involved in bonded labour or prostitution. Girls were more often involved in labour activities than boys (66.8% versus 62.1%, p = 0.048): this difference was most obvious with street trading (p = 0.0004). Most of the children (82.2%) involved in labour activities did so on the instruction of one or both parents in order to contribute to family income. Children of parents with low socio-economic status or of poorly educated parents were significantly involved in labour activities (p = 0.01 and p = 0.001 respectively). Child labour was also significantly associated with increasing number of children in the family size (p = 0.002). A higher prevalence rate of child labour was observed among children living with parents and relations than among those living with unrelated guardians.

**Conclusion:**

It is concluded that smaller family size, parental education and family economic enhancement would reduce the pressure on parents to engage their children in labour activities.

## Background

Child labour covers all economic activities carried out by children regardless of their occupational status [1]. It has probably being in existence almost as long as the history of mankind. Economic activity is a broad concept that encompasses most productive activities of children. It includes both work that is permissible under the International Labour Organization's (ILO) conventions and that which is not [2]. Child labour is prevalent worldwide, occurring both in developing and developed countries [3,4]. It is estimated that about 352 million children are engaged in some form of economic activity in the world [2]. Estimates of the ILO put the number of children fully at work in developing countries at 120 million and those working and schooling at 250 million [5].

Most of child labour takes place in Asia, the Pacifics and Africa [6-9]. In most parts of Africa the prevalence ranges from 20% to 54% [10]. The prevalence in Nigeria, Cote d'Ivoire and Zambia ranges from 20% to 30% [10]. Very few Nigerian studies [6] provide information on children who both school and work. Some studies [11] from other parts of the world actually excluded schooling children from the definition of child labour. This study aims to document the prevalence and types of child labour among primary and junior secondary school pupils in Nigeria.

## Methods

This presentation is part of a broader study on child labour among school children in public primary and junior secondary schools in Sagamu Local Government Area of Ogun State, Nigeria. The study was conducted between October 1998 and September 1999 (inclusive). It was approved by the Ethical Committee of the Olabisi Onabanjo University Teaching Hospital Sagamu (OOUTH).

Sagamu Local Government Area (SLGA) is one of the 15 Local Government Areas in the State. It is predominantly rural and semi-urban with a land space of 68.4 sq km. The estimated population for the year 1997 is 177,514. Administratively, SLGA is made up of 11 wards. The total school enrolment in the 50 primary and 16 junior secondary schools (JSS) for the year 1997 was 30,597, comprising 21,476 primary and 9,121 JSS pupils. Excluding 6,762 new entrants who did not have academic records for the preceding year left 23,835 pupils (17,891 primary and 5,944 junior secondary school pupils). Thus the ratio of primary to junior secondary school pupil population was about 3 to 1. This ratio was considered while selecting the subjects for the study.

### Sampling

The survey sample was drawn randomly from all 50 public primary and 16 secondary schools in the LGA. Using ballot papers, one primary and one secondary school were randomly selected from each administrative ward. However, one ward had no primary school and four wards had no secondary school. Thus, a total of ten primary and seven secondary schools were selected. There were five classes in each primary school and two in each junior secondary after excluding primary 1 and JSS1 pupils because they were new entrants in their respective schools and therefore did not have academic records for the preceding year. Each class was made up of at least two arms.

One arm of each eligible class in the selected schools was randomly selected, also using ballot papers. Altogether, 64 classes were selected: 50 classes from 10 primary schools and 14 classes from 7 secondary schools. Thirty pupils were randomly selected from each class to form the study school. The pupils were interviewed one at a time between the hours of 8.00 am and 2.00 pm on Monday through Friday using a structured, close-ended questionnaire. The interviews were conducted by author MBF with the help of previously trained research assistants. Relevant background information on age, sex, family background, involvement or otherwise in after-school economic activities were obtained. Information on parental education, occupation/income was obtained through requests accompanying letters to parents and guardians asking for their consent. The occupation/income and educational attainments of the parents were used to determine socio-economic index scores of the children using modified criteria described by Oyedeji [12].

Each parent was given two index scores: one for occupation/income and the other for educational attainment. The scores were rated on a scale of 1 to 5, from the more educated and more highly placed occupation/income groups to the least advantaged. The mean of the four scores for both parents was calculated and the value became the assigned socioeconomic group of the family/child. Where the calculated mean was not a whole number, the next higher integer was used.

Child labour was defined as any type of economic task, paid, unpaid, or exploitative, engaged in by a child less than 18 years of age, which places the interests of the beneficiary well above those of the child and is detrimental to the physical mental, social, educational and moral development of the child [1].

For the purpose of analysis the study children were grouped into two; those involved in child labour and those who were not. Chi-square analysis was used to compare proportions and probability; (p) values less than 5% (0.05) were accepted as statistically significant.

## Results

**Figure 1:**
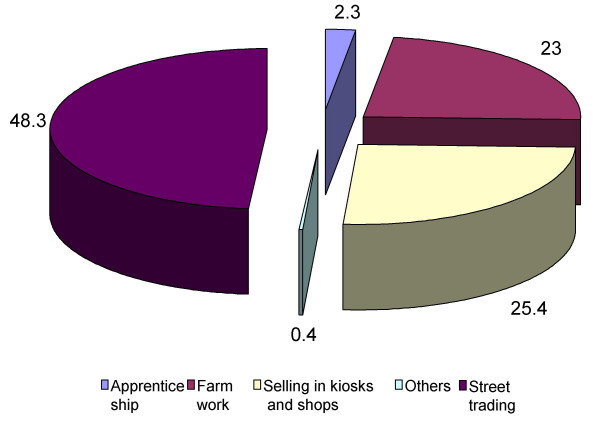
Pattern of child labour among study children.

A total of 1675 day pupils were recruited into the study. The male/female ratio was 1:1.02. The majority (86.4%) of respondents were of Yoruba extract. Their ages ranged between 5 and less than 18 years. Those in primary schools were 1299 while 376 were in junior secondary schools as shown in Table 2. One thousand and eighty pupils were involved in child labour giving an overall prevalence of 64.5%. The leading occupational activity was street trading (Figure 1). Three hundred and eighty seven girls were engaged in street trading compared to 308 boys (45.7% versus 37.2 %, X = 0.004). No child was involved in bonded labour or prostitution.

**Table 1:** General characteristics of the subjects (n = 1675).

***Subjects***	**No. of respondents (%)**
**Sex distribution**	
Male	828 (49.4)
Female	847 (50.6)
**Tribe**	
Yoruba	1448 (86.4)
Igbo	111 (6.60)
Hausa	35 (2.1)
Others	81 (4.9)
**Age distribution**	
5 to 6 years	50 (3.0)
7 to 16 years	1594 (95.2)
17 years	31 (1.8)
**Level of education**	
Primary	1299 (77.6)
Secondary	376 (22.4)

**Table 2:** Distribution of subjects involved in child labour by gender, education, family religion, custodian and number of children in the family. (n = 1675)

	Involved in child labour	Not involved in child labour	Total		
Characteristics	no (%)	no (%)		χ^2^	p-value
**Gender**					
Male	514 (62.1)	314 (37.9)	828 (100.0)		
Female	566 (66.8)	281 (33.2)	847 (100.0)	3.91	0.048
**Level of schooling**					
Primary	891 (68.6)	409 (31.4)	1299 (100.0)		
Secondary	189 (50.3)	187 (49.7)	376 (100.0)	42.8	< 0.0001
**Religion**					
Christianity	631 (61.9)	388 (38.1)	1019 (100.0)		
Islam	445 (68.3)	207 (31.7)	652 (100.0)	6.94	0.008*
Others	4 (100.0)	0 (0.0)	4 (100.0)		
**Custodian**					
Both parents	643 (65.8)	334 (34.2)	977 (100.0)		
Single father	73 (54.1)	62 (45.9)	135 (100.0)		
Single mother	196 (65.8)	102 (34.2)	298 (100.0)		
Other relatives	165 (64.7)	90 (35.3)	255 (100.0)		
Unrelated guardian	3 (30.0)	7 (70.0)	10 (100.0)	12.74	0.014
**No. of children in the family**					
1	13 (46.4)	14 (53.6)	27 (100.0)		
2 or 3	143 (58.6)	101 (41.4)	244 (100.0)		
4 or 5	352 (62.7)	209 (37.3)	561 (100.0)		
6 or 7	308 (68.6)	142 (31.4)	450 (100.0)		
8 or 9	264 (67.2)	129 (32.8)	393 (100.0)	9.13	0.0025#

*Comparison was limited to Christians and Moslems because there were very few practitioners of other religions.

# Chi-square analysis for linear trend

**Figure 2:**
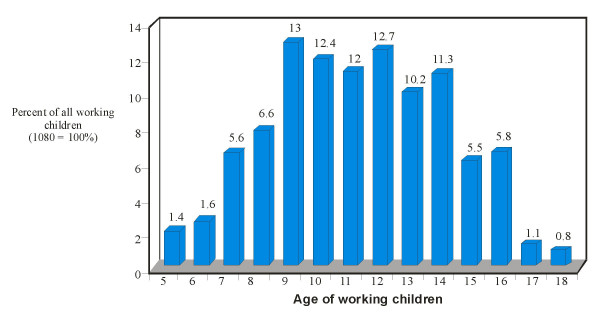
Age distribution of children involved in child labour.

As shown in Figure 2, child labour was most prevalent among children 9 to 14 years of age (10.2% to 13.0% of all the children involved in child labour). Table 2 shows the distribution of the children according to gender, educational level, family religion, custodian and number of children in the family. Girls, primary school pupils and Moslem children were all significantly involved in child labour than their counterparts (p = 0.048, 0.001 and 0.008 respectively). Comparison was limited to Christians and Moslems because there were very few practitioners of other religions.

Over 80% of the study children lived with one or both parents, 15.2% with other relatives and very few (0.3%) with unrelated guardians. Children living with parents (both or single) and relatives had higher prevalence rates of child labour. The number of children living with unrelated guardians was relatively small but this group had less than 50% the prevalence of child labour of their counterparts living with relatives (χ^2 ^= 5.22, p <0.025). The prevalence of child labour did not vary significantly among children living with different types of relatives (p > 0.05). A significant rising trend of involvement in child labour with increasing number of children in the family (χ^2 ^for trend = 9.13, p = 0.0025) was observed.

As many as 82.8% of working children were assigned economic tasks by one or both parents. Also 89.8% of the children performed these tasks in order to contribute to the family economy for feeding and for school fees.

In Table 3 child labour decreased with increasing parental education. This trend was clearer with respect to maternal education. However, in Table 4, child labour rates increased with decreasing parental socio-economic status.

**Table 3:** Distribution of subjects involved in labour activities according to parental education.

	Father		Mother	
**Parental level of education**	no (%)	**Odds ratio**	no (%)	**Odds ratio**
University graduate or equivalent	30 (35.7)	1.00	14 (45.2)	1.00
Senior secondary school certificate/Teaching or other professional certificate	107 (54.6)	2.16	72 (46.2)	1.04
Junior secondary school certificate/Teacher's Grade II certificate/Equivalent	136 (68.7)	3.95	86 (54.8)	1.47
Junior secondary school education/Modern III certificate/Primary school leaving certificate	513 (68.5)	3.91	516 (68.3)	2.62
No formal education/Quranic school/Barely literate/dead parent	227 (67.2)	3.68	329 (68.1)	2.59
χ^2 ^for trend	29.33		32.86	
p	< 0.00001		< 0.00001	

**Table 4:** Distribution of subjects according to parental socio-economic status.

Socioeconomic class	Involved in child labour n (%)	Not involved in child labour n (%)	Total respondents	Odds ratio
I	18 (32.7)	37(67.3)	55	1.00
II	78 (50.0)	78 (50.0)	156	2.06
III	403 (64.5)	222(35.5)	625	3.73
IV	442 (70.4)	186(29.6)	628	4.88
V	42 (70%)	18 (30%)	60	4.8

χ^2 ^for trend = 40.87, p < 0.00001

OR = Odds ratio

I = very high

II = high

III = medium

IV = low

V = very low

## Discussion

Child labour is a topical issue of global concern but statistics on the subject are often underestimated partly because of practical difficulties and also because of differences in the design and implementation of surveys [5].

The observed prevalence rate of 64.5% found in this study confirms the existence of child labour as an important secondary activity of school children in Sagamu Local Government Area of Nigeria. It is in consonance with high rates reported in Nigeria [10] and other developing countries [7]. The finding is also in keeping with an earlier report from Nigeria [6] and various parts of the world, that children both school and work [4,9,13]. Results of surveys by the ILO in four developing countries found that two-thirds of children combined school with work [4,15]. A rate of 51.9% was found in an earlier study carried out in Nigeria [16], while the rate was about 50 % in both Cairo and Bogotá [17]. The differences in prevalence rate may reflect differences in methodology and data collection.

The present study focused on children within the compulsory age of schooling, which is also probably the peak age for child labour. Also, this study unlike previous local ones was school-based. The children were the primary respondents and interviews were conducted away from the influence or interference of parents.

There was a higher representation of girls than boys among working children also in accordance with previous observations [7,12]. It is attractive to conjecture that the finding reflects gender bias in upbringing aimed at preparing the girls for traditional roles of small-scale economic trades to boost family economy. It may also be speculated that girls in the traditional African society are more amenable and responsive to parental control and as such are assigned economic tasks more often. The present study considered the role of children in economic tasks within the family enterprise and in and around the home. These activities usually involve more girls than boys in the traditional Nigerian setting.

Moslem children were more often involved in child labour than their Christian counterparts. Other studies [19,20] had noted the influence of religious affiliation in the Moslem states of northern Nigeria and commented that wife seclusion (purdah) often puts a great economic burden on children. However, the practice of purdah is not a feature of southern Nigeria Islamism. Thus, the findings cannot be explained on that basis. It may however be speculated that the association of Moslem religion with child labour is related to family size.

There was an increasing trend of engagement in child labour with increasing number of children within the family. This observation is consistent with other studies [21]. It may be, as has been suggested by other workers [22], that the desire for large family size is based on potential economic considerations. On the other hand, it may be validly argued, that a large family size, with the attendant spread of lean resources over many dependants, would force parents to engage their children in labour activities.

Street trading was the dominant economic activity of working children in the study, agreeing with the findings from other urban and semi-urban settings in Nigeria [16,17]. However in Enugu [23], elsewhere in the country house servants constitute about two-thirds of the work force. Enugu is a major regional headquarters and is predominantly inhabited by the Igbo tribe in contrast to Sagamu LGA with its rural/semi-urban status and predominantly Yoruba population. The social and economic pressures differ and may account in part, for the variations in major economic tasks performed by children.

It is comforting that, in contrast to reports from some other developing countries [3,18,24], no child in the present series was bonded or involved in prostitution. These practices are strange to the Ijebu Yoruba, buttressing the well-known influence of culture and tradition [10] on prevalence of child labour. This influence of culture, with respect to child rearing might also partly explain the high prevalence rate of child labour herein reported. The Ijebu Yoruba are known to be very industrious. They get their children introduced to family economic activities rather early.

The stress of rural and semi-urban economies might also have put pressure on parents to engage their children in some form of economic activity in order to enhance family income [3,7,13,21,25-27].

Most working children were assigned their roles by their parents, largely to supplement family income. The prevalence of child labour did not differ significantly among children living with one or both parents or with a related guardian, Children living with single mothers were, not surprisingly, also more affected. Harsh economic realities, the burden of paying school fees and catering for a large family size may be the underlying reason parents compel their children to engage in work activities. On the other hand, children living with non-related guardians were surprisingly significantly less involved in labour activities. This finding however should be interpreted with a lot of caution because the number of children living with non-relatives was small. It is noteworthy however, to the extent that children living with their biological parents should theoretically be better cared for than those in foster homes. It is conceivable, that affected children may have been sent to live with more affluent guardians to give such children better prospects for the future.

The prevalence of child labour increased with decreasing parental education and socio-economic class. These findings agree with other studies [7,8,13,21,28]. The reasons for these observations are fairly obvious. Educated parents are more likely to understand the need for a growing child to concentrate on activities capable of enhancing realization of their full potential and the need to avoid potentially 'harmful' activities. Also, better socio-economic standing removes the pressure on parents to put their children to work.

## Conclusion

There is a need to protect the girl child, advocate reduction in family size, and promote parental education/economic empowerment in order to reduce the urge on children to perform economic roles. There is also a need to study all the ramifications of child labour for proper planning and for the protection of school children if Nigeria is to improve on her child survival, developmental and protection indices in the near future.

## Competing interests

The author(s) declare that they have no competing interests.

## Authors' contributions

MBF conceived of the study, participated in its design, collection, statistical analysis and interpretation of data. FON contributed to the statistical analysis, interpretation of the data and has been involved in drafting and revising the manuscript.

AOO participated in coordination of the study and revision of the manuscript. All authors read and approved the final manuscript.

## Pre-publication history

The pre-publication history for this paper can be accessed here:


